# Prognostic role of lipoprotein(a) in atherosclerotic cardiovascular disease risk from a perspective on current risk stratification

**DOI:** 10.1002/mco2.773

**Published:** 2024-10-31

**Authors:** Sha Li, Hui‐Hui Liu, Yan Zhang, Meng Zhang, Hui‐Wen Zhang, Cheng‐Gang Zhu, Na‐Qiong Wu, Rui‐Xia Xu, Qian Dong, Jie Qian, Ke‐Fei Dou, Yuan‐Lin Guo, Jian‐Jun Li

**Affiliations:** ^1^ Cardiometabolic Center State Key Laboratory of Cardiovascular Disease FuWai Hospital National Center for Cardiovascular Diseases Chinese Academy of Medical Sciences Peking Union Medical College Beijing China

**Keywords:** atherosclerotic cardiovascular disease, Chinese, lipoprotein(a), risk stratification

## Abstract

Lipoprotein(a) [Lp(a)] is an emerging predictor for atherosclerotic cardiovascular disease (ASCVD) but the association from a perspective on current risk stratification was unknown. A cohort of 9944 Chinese patients with ASCVD was recruited and refined into very‐high‐risk (VHR) and non‐VHR subgroups according to current guideline. Lp(a) plasma levels were divided by its concentration (<30, 30–50, 50–75, and ≥75 mg/dL) and percentile zones (<25th, 25–50th, 50–75th, 75–90th, ≥90th). Cardiovascular events (CVEs) occurred during an average of 38.5 months’ follow‐up were recorded. We found that Lp(a) was increased with risk stratification of ASCVD increasing. Prevalence of CVEs had a significantly increasing trend with gradients of Lp(a) elevation in VHR but not in non‐VHR subgroup. The adjusted HRs (95%CIs) for CVEs were 1.75(1.25–2.46) in the highest group of Lp(a) ≥75 mg/dL compared with the group of Lp(a) <30 mg/dL as the reference in overall patients, 2.18(1.32–3.58) in VHR subgroup and 1.43(0.93–2.18) in non‐VHR subgroup, respectively. The adjusted HRs (95%CIs) at the highest grade of Lp(a) levels (≥90th) were 1.72(1.19–2.50) in overall population, 2.83(1.53–5.24) in VHR subgroup and 1.38(0.86–2.12) in non‐VHR subgroup, respectively. These findings suggested that Lp(a) might contribute more to CVEs risk in VHR subgroup of ASCVD.

## Graphical abstract

We assessed the relationship of current guideline‐stratification of ASCVD with plasma Lp(a). Patients at higher grade of risk stratification showed higher Lp(a) level. Participants with both high grades of Lp(a) and risk stratification were at significantly high risk for recurrent CVEs. The additional value of Lp(a) elevation for CVEs risk prediction was evident in patients at VHR but not those at non‐VHR

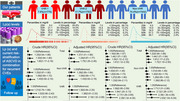

## 1 INTRODUCTION

Since the mid‐1990s, the advent of genetic studies, particularly by means of Mendelian randomization, offered robust evidence for a causal role of high lipoprotein(a) [Lp(a)] levels in cardiovascular diseases (CVD).^1^, ^2^, ^3^ Studies of Lp(a) in atherosclerotic CVD (ASCVD) and cardiovascular events (CVEs) have expanded substantially.^4^, ^5^, ^6^ These supports prompted expert appraisal and evaluation of Lp(a) in clinical guidelines.^7^, ^8^, ^9^, ^10^ The 2010 European Atherosclerosis Society (EAS) consensus statement on Lp(a) suggested increased Lp(a) as a cardiovascular risk factor and recommended circulating Lp(a) measurement in a selected subjects including those intermediate‐ to high‐risk patients for risk stratification.^7^ The latest EAS statement has been updated with a focus on Lp(a), catalyzing a worldwide effort to understand the underlying causes of variability of Lp(a) and its association with ASCVD across various ancestry populations.^8^ The stage has aroused growing attention and is now truly set for Lp(a) as the revenant.^1^ However, there are considerable racial variation in Lp(a) concentrations and the population level distribution, confusing questions regarding this mystery lipoprotein involved in the Lp(a) assay, the potential subjects to be analyzed, the elevated risk threshold, and so forth, particularly in China.^10^

ASCVD is an ancient disease with an enormous worldwide impact. Ongoing efforts have made to improve our knowledge and its diagnosis and treatment strategies. ^11^ It has been well established that there is a heterogeneous pattern of recurrence and adverse outcomes between subjects with documented ASCVD. Historically, patients at very‐high‐risk (VHR) were comprised of those who had a proven ASCVD or a risk equivalent using a “blanket” approach in risk stratification.^11^ The current paradigm of guidelines first refines patients with ASCVD into VHR and non‐VHR subgroup.^12^, ^13^ Emerging evidence strongly indicate that the refinement is essential and patients of VHR subgroup deserves a more veritable approach in clinical practice.^14^, ^15^ For example, the higher the baseline risk, the more likely the patient is to benefit from statin treatment.^16^

The recent investigations have addressed the residual risk driven by Lp(a) in patients with potent statin therapy.^17^ It has been hypothesized that Lp(a) could be a useful index for identifying patients in which the atherosclerotic process is more advanced.^18^, ^19^ However, Lp(a) is requested to measure in just a small minority of patients with ASCVD in real world.^20^ Additionally, an extensive literature review on Lp(a) in China has been conducted, indicating that it is prevalent and unique in CVD regulation.^10^ The role of Lp(a) across the current different risk‐stratification subgroups of ASCVD in CVEs prediction remains unknown. Therefore, in the present study, we measured consecutively Lp(a) plasma levels among a Chinese cohort of documented ASCVD and aimed to examine the relationship of Lp(a) levels with CVEs from a prospective on current guideline‐refined risk stratification.

The refined risk stratification of ASCVD was defined according to the 2018 American Heart Association/American College of Cardiology cholesterol guidelines.^12^ Patients in VHR subgroup were defined as a history of ≥2 major ASCVD events (VHR‐1) or 1 major event and ≥2 high‐risk conditions (VHR‐2). Other subjects with ASCVD were defined as non‐VHR. Major ASCVD events were most recently diagnosed with acute coronary syndrome (ACS) in the last 12 months, a previous myocardial infarction (MI) except for ACS, a history of ischemic stroke or symptomatic peripheral arterial disease. The high‐risk conditions were those who were older than 65 years of age, those with familial hypercholesterolemia (FH), those who had undergone coronary artery bypass grafting or percutaneous coronary intervention outside of the major ASCVD event(s), those with diabetes mellitus (DM), those with hypertension, those with chronic renal disease (estimated glomerular filtration rate, eGFR 15–59 mL/min/1.73 m^2^), those who had been smokers, those who had continued to have high low‐density lipoprotein (LDL) cholesterol (LDL‐C) levels (LDL‐C > 100 mg/dL) despite maximally tolerated statin therapy, or who had a history of congestive heart failure.

## 2 RESULTS

### 2.1 Association of Lp(a) with ASCVD risk stratification

**FIGURE 1:**
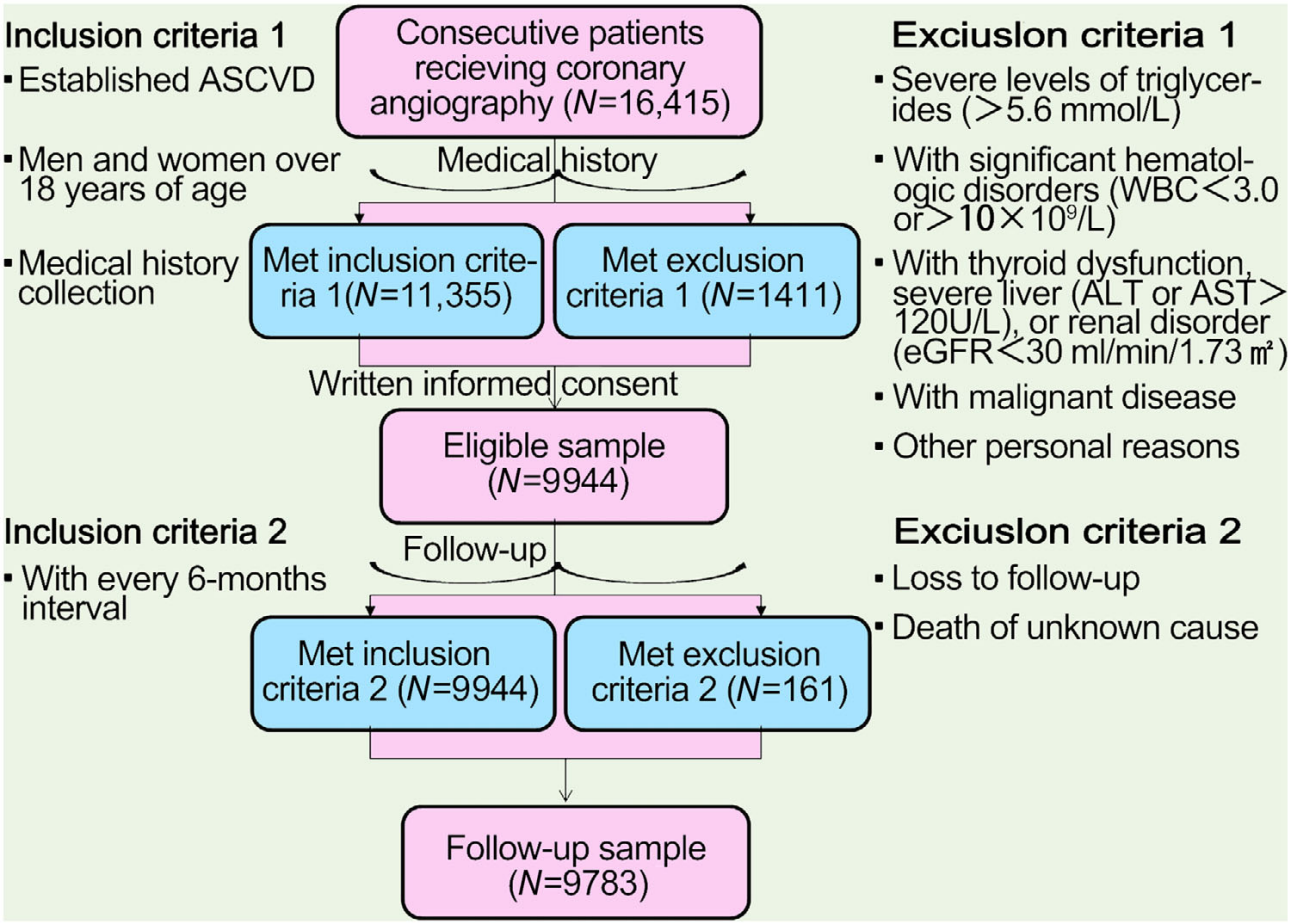
The flowchart of the present study. ASCVD, atherosclerotic cardiovascular disease; WBC, white blood cell; ALT, alanine aminotransferase; AST, aspartate aminotransferase; eGFR, estimated glomerular filtration rate.

**FIGURE 2:**
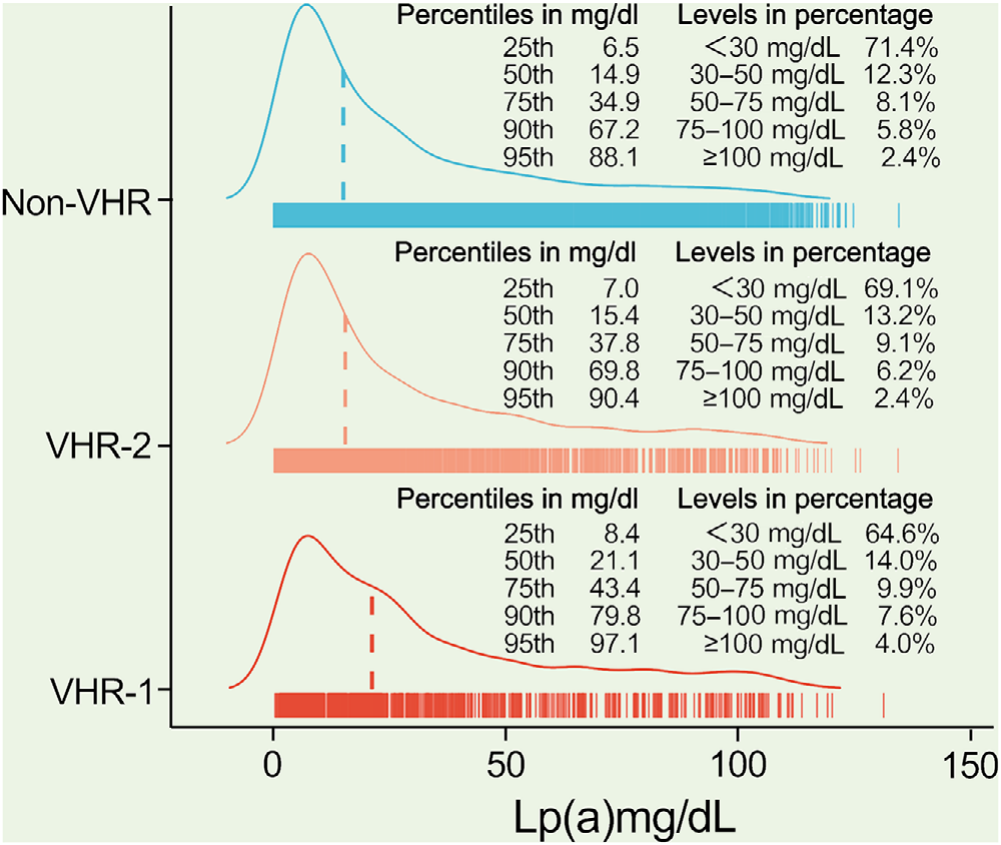
Distribution of Lp(a) levels in each group according to current risk stratification of ASCVD. Percentages of the population with Lp(a) of <30, 30−50, 50−75, 75−100, and ≥100 mg/dL, and values of Lp(a) according to percentiles (25th, 50th, 75th, 90th, and 95th), respectively. Lp(a), lipoprotein(a); VHR, very‐high‐risk.

The flowchart illustrating the enrollment process and follow‐up procedures was shown in Figure 1. A total of 9944 adults with confirmed ASCVD were enrolled at baseline. After all, the record was collected from 9783 patients, with 407 patients with CVEs and 9376 without CVEs during an average of 38.5 months’ follow‐up. Patients with CVEs recorded during the follow‐up had higher levels of Lp(a) [21.2(9.9–44.6) vs. 15.3(6.6–35.6) mg/dL], numbers of major ASCVD events (0.6 ± 0.8 vs. 0.4 ± 0.6), and high‐risk conditions (2.4 ± 1.2 vs. 2.0 ± 1.1) at baseline when compared with those without recurrent events (Table 1). As shown in Figure 2, plasma Lp(a) levels had a skewed distribution with a tail toward the highest levels in each refined group of ASCVD risk‐stratification. There was an ascending gradient regarding the percentiles and concentrations of Lp(a) across refined groups with increasing risk stratification. Specially, the highest median (interquartile range, IQR) of Lp(a) was found in patients at VHR‐1 was 21.1(8.4–43.4) mg/dL, followed by 15.4(7.0–37.8) mg/dL in those at VHR‐2 and 14.9(6.5–67.2) mg/dL in those at non‐VHR. The proportions of patients with elevated Lp(a), defined as a level ≥30 mg/dL (or high ≥50 mg/dL), in patients at VHR‐1, VHR‐2, and non‐VHR were 35.4% (21.5%), 30.9% (17.7%), and 28.6% (16.3%), respectively.

**TABLE 1:** Clinical characteristics of the study patients according to CVEs.

	Total (*n* = 9944)	With CVEs (*n* = 407)	Without CVEs (*n* = 9376)	*p* for trend
Clinical characteristics				
Male, %(*n*)	73.1 (7268)	70.0 (285)	73.4 (6886)	0.137
Age (y)	57.8 ± 10.3	61.9 ± 9.7	57.5 ± 10.2	**<0.001**
BMI (kg/m^2^)	25.9 ± 3.2	25.7 ± 3.1	26.0 ± 3.2	0.133
SBP (mmHg)	126.5 ± 17.1	127.7 ± 18.2	126.4 ± 17.0	0.155
DBP (mmHg)	77.7 ± 10.7	76.4 ± 10.6	77.7 ± 10.7	**0.024**
TG (mmol/L)	1.5 (1.1‐2.1)	1.5 (1.1‐2.1)	1.5 (1.1‐2.1)	0.923
LDL‐C (mmol/L)	2.5 ± 1.0	2.6 ± 1.0	2.5 ± 1.0	0.364
HDL‐C (mmol/L)	1.1 ± 0.3	1.1 ± 0.3	1.1 ± 0.3	0.894
Lp(a) (mg/dL)	15.5 (6.7–36.0)	21.2 (9.9–44.6)	15.3 (6.6–35.6)	**<0.001**
Glucose (mmol/L)	6.0 ± 1.8	5.9 ± 1.9	6.0 ± 1.8	0.743
HbA1C (%)	6.4 ± 1.1	6.6 ± 1.3	6.4 ± 1.1	**<0.001**
Creatinine (µmol/L)	78.9 ± 21.2	80.2 ± 18.9	78.8 ± 21.3	0.192
Current smoking, %(*n*)	37.5 (3730)	31.2 (127)	38.0 (3559)	**0.007**
Hypertension, %(*n*)	64.7 (6429)	69.8 (284)	64.3 (6033)	**0.025**
Diabetes, %(*n*)	33.2 (3302)	39.6 (161)	32.9 (3085)	**0.005**
Number of major ASCVD events	0.4 ± 0.6	0.6 ± 0.8	0.4 ± 0.6	**<0.001**
Number of high‐risk conditions	2.0 ± 1.1	2.4 ± 1.2	2.0 ± 1.1	**<0.001**
Medications at enrollment				
Antiplatelet drugs, %(*n*)	64.1 (6371)	49.4 (201)	64.7 (6066)	**<0.001**
ACEI/ARB, %(*n*)	18.8 (1871)	12.3 (50)	19.2 (1799)	**<0.001**
β‐Blockers, %(*n*)	36.2 (3595)	27.5 (112)	36.5 (3423)	**<0.001**
Statins, %(*n*)	72.2 (7178)	62.7 (255)	72.8 (6824)	**<0.001**

*Note*: Data shown are %(*n*), mean ± SD, or median (IQR). *p* Values are shown for trend.

Abbreviations: ASCVD, atherosclerotic cardiovascular disease; ACEI, angiotensin converting enzyme inhibitors; ARB, angiotensin receptor blocker; BMI, body mass index; CVE, cardiovascular event; DBP, diastolic blood pressure; HDL‐C, high‐density lipoprotein cholesterol; HbA1C, hemoglobin A1C; LDL‐C, low‐density lipoprotein cholesterol; Lp(a), lipoprotein(a); SBP, systolic blood pressure; TG, triglycerides.

The bold values indicate statistical signifcance and are bolded to improve the readability of the table.

### 2.2 Prevalence of CVEs in different risk stratification and Lp(a) levels

**FIGURE 3:**
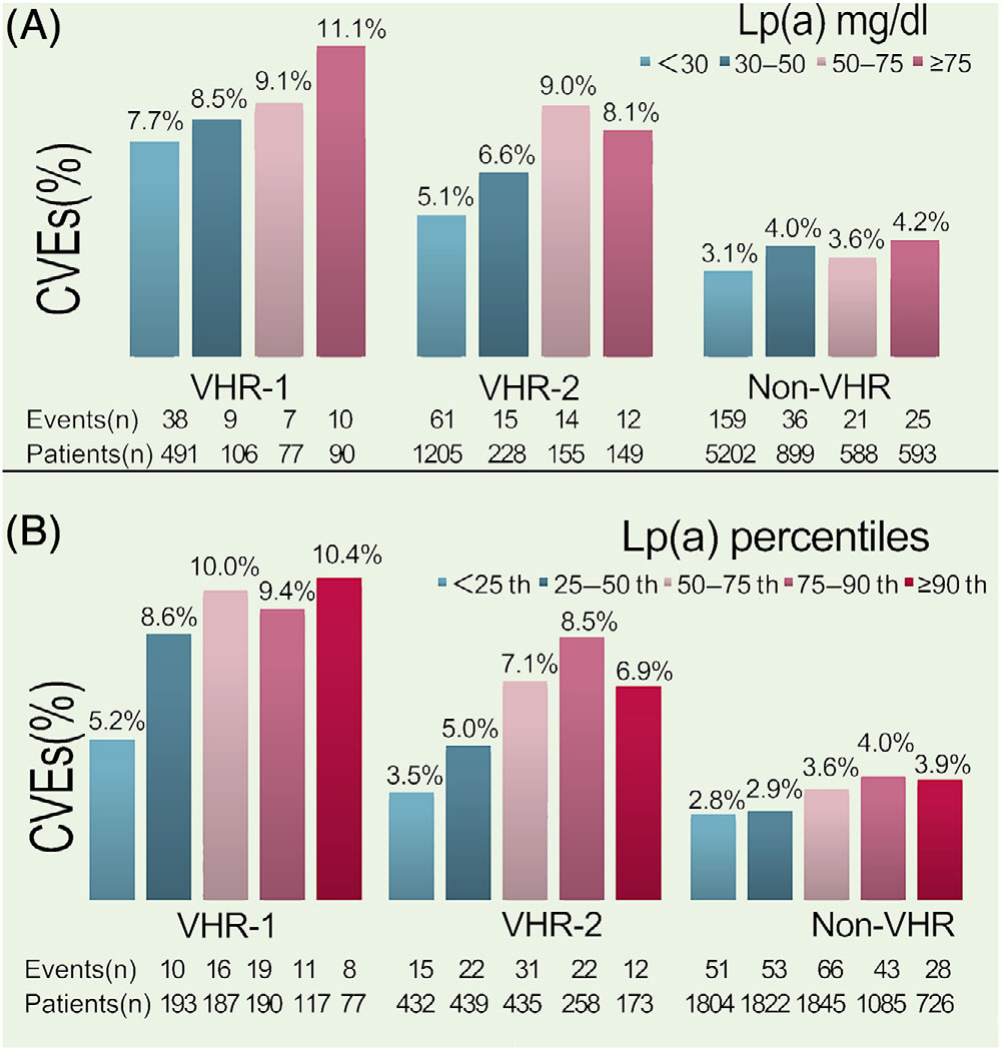
Proportions of CVEs in different risk‐stratification group across Lp(a) distribution. (A) Lp(a) stratified by its concentration (<30, 30−50, 50−75, and ≥75 mg/dL). (B) Lp(a) stratified by its percentile zones (<25th, 25−50th, 50−75th, 75−90th, and ≥90th). CVE, cardiovascular event; Lp(a), lipoprotein(a); VHR, very‐high‐risk.

Prevalence of CVEs in each refined group of ASCVD risk stratification had an increasing trend with gradients of Lp(a) elevation evaluated by its concentrations or percentiles (Figure 3). However, the trend was evident in patients at VHR but not statistically significant in those at non‐VHR. The prevalence of CVEs in patients at VHR‐1, VHR‐2, and non‐VHR were 11.1 versus 9.1 versus 8.5 versus 7.7%, 8.1 versus 9.0 versus 6.6 versus 5.1%, and 4.2 versus 3.6 versus 4.0 versus 3.1% when compared among different gradients of Lp(a) concentrations (≥75 vs. 50–75 mg/dL vs. 30–50 vs. <30 mg/dL). Also, we found the similar prevalence in those three groups among different gradients of Lp(a) percentiles (≥90th vs. 75−90th vs. 50−75th vs. 25−50th vs. < 25th, VHR‐1 10.4 vs. 9.4 vs. 10.0 vs. 8.6 vs. 5.2%, VHR‐2 6.9 vs. 8.5 vs. 7.1 vs. 5.0 vs. 3.5%, non‐VHR 3.9 vs. 4.0 vs. 3.6 vs. 2.9 vs. 2.8%).

### 2.3 Association of Lp(a) and CVEs at different risk stratification

**FIGURE 4:**
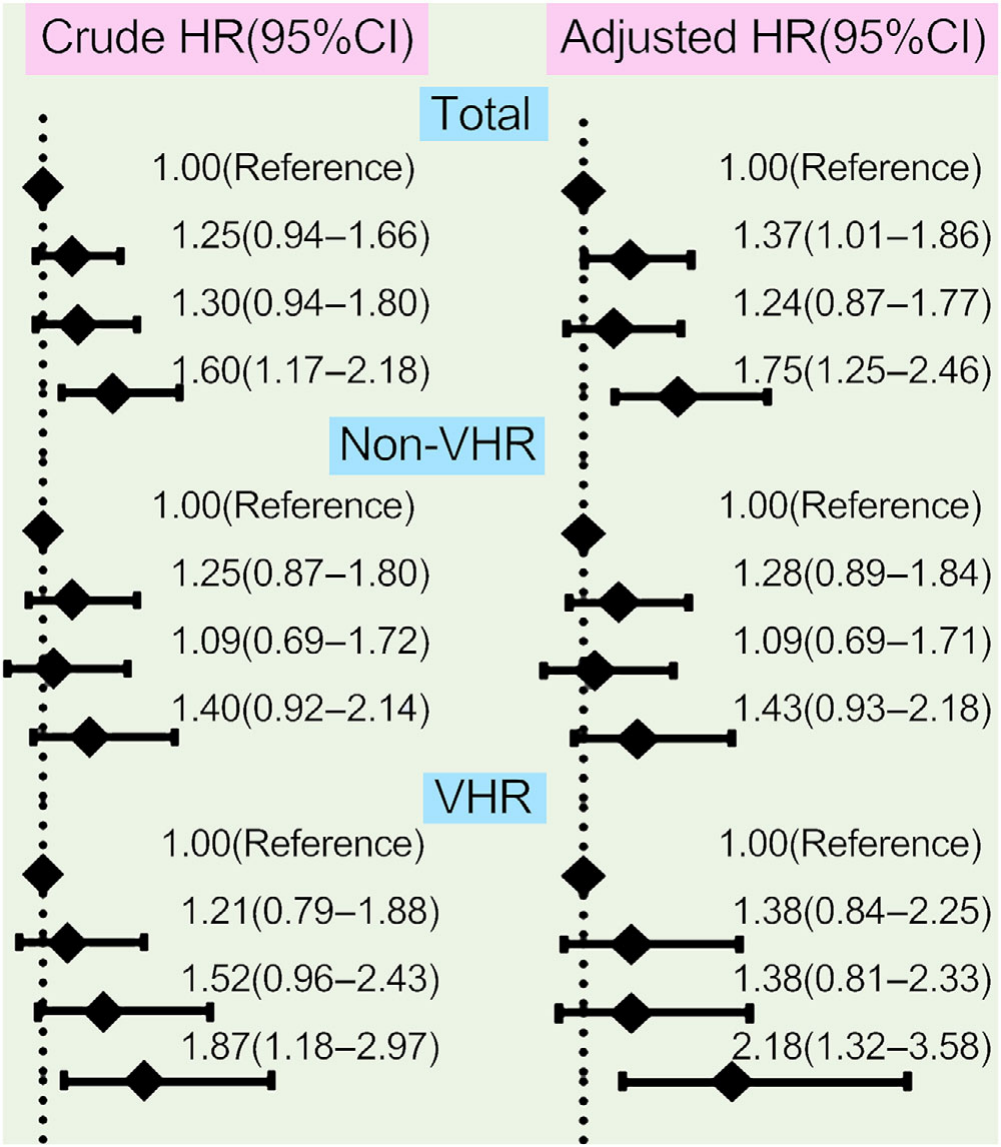
Values of Lp(a) evaluated by its concentrations to current risk stratification in CVEs prediction. Lp(a) plasma levels were divided by its concentration (<30, 30−50, 50−75, and ≥75 mg/dL). Univariate and multivariate Cox proportional hazards regression analyses were performed. Multivariate models adjusted for age, sex, BMI, SBP, DM, LDL‐C levels, current smoking, and medications at enrollment. CVE, cardiovascular event; CI, confidence interval; HR, hazard ratio; Lp(a), lipoprotein(a).

**FIGURE 5:**
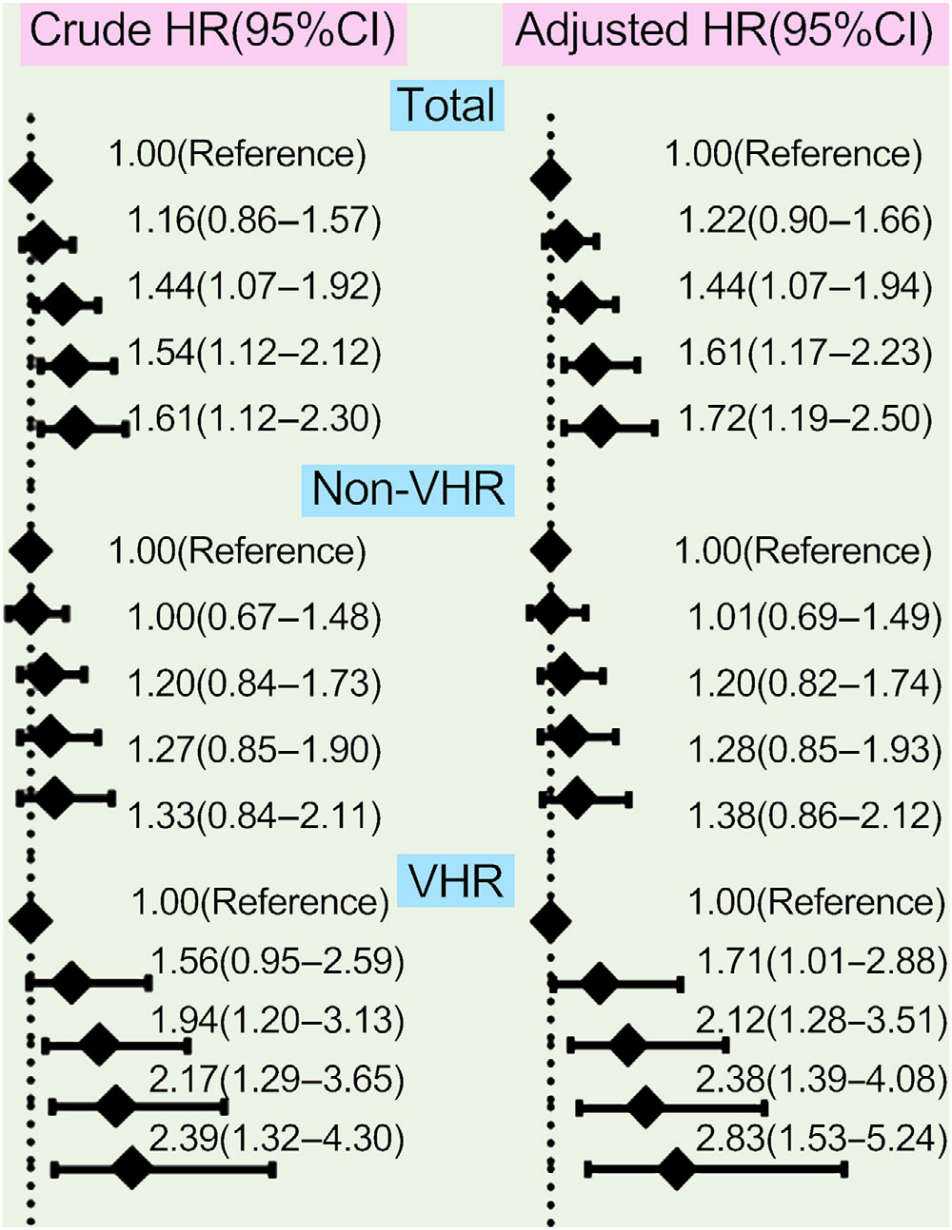
Values of Lp(a) evaluated by its percentiles to current risk stratification in CVEs prediction. Lp(a) plasma levels were divided by its percentile zones (<25th, 25−50th, 50−75th, 75−90th, and ≥90th). Univariate and multivariate Cox proportional hazards regression analyses were performed. Multivariate models adjusted for age, sex, BMI, SBP, DM, LDL‐C levels, current smoking, and medications at enrollment. CVE, cardiovascular event; CI, confidence interval; HR, hazard ratio; Lp(a), lipoprotein(a).

The analyses of the association between different concentrations of Lp(a) and CVEs were performed among the 9783 patients who completed the follow‐up in this study, which had been observed separately in the overall population, non‐VHR, and VHR subgroup in Figures 4 and 5. Patients at the highest grade of Lp(a) levels appeared at the highest risk for CVEs in overall population and VHR subgroup but no such significant association was observed in patients with non‐VHR. The crude hazard rations (HRs) (95% confidence intervals [CIs]) were 1.60(1.17–2.18) in the highest group of Lp(a) ≥75 mg/dL compared with the group of Lp(a) <30 mg/dL as the reference in overall patients, 1.87(1.18–2.97) in VHR subgroup and 1.40(0.92–2.14) in non‐VHR subgroup, respectively. Moreover, the adjusted HRs (95%CIs) were 1.75(1.25–2.46) in the highest group of Lp(a) ≥75 mg/dL compared with the group of Lp(a) <30 mg/dL as the reference in overall patients, 2.18(1.32–3.58) in VHR subgroup and 1.43(0.93–2.18) in non‐VHR subgroup, respectively (Figure 4). The above scenario was similar when evaluated Lp(a) levels stratified by its percentiles in each refined group of ASCVD risk stratification (Figure 5). The adjusted HRs (95%CIs) at the highest grade of Lp(a) levels (≥90th) were 1.72(1.19–2.50) in overall population, 2.83(1.53–5.24) in VHR subgroup, and 1.38(0.86–2.12) in non‐VHR subgroup, respectively. Likewise, the HRs of Lp(a) elevation with CVEs showed a significantly increasing trend in both overall population and VHR subgroup but not in non‐VHR subgroup.

Kaplan–Meier survival curves compared the cumulative incidence of CVEs stratified by the Lp(a) levels stratified by its percentiles at different risk stratification of ASCVD were shown in Figure S1. An incrementally higher Lp(a) was associated with an increasingly higher cumulative incidence of CVEs in overall population (*p* = 0.018) and VHR subgroup (*p* = 0.012) but not in non‐VHR subgroup (*p* = 0.497). Meanwhile, we performed restricted cubic spline (RCS) of Lp(a) and CVEs risk, suggesting that the risk of recurrent events might increase with elevating Lp(a) levels in those patients at VHR but not in those at non‐VHR (Figure S2). Furthermore, the compared receiver operator characteristic (ROC) curves showed a better discrimination for Lp(a) in VHR (C‐statistics 0.585) than non‐VHR (C‐statistics 0.544), that internally validated to divide the training and validation cohort presented the similar results (Figure S3). In addition, we compared the values of Lp(a) and LDL‐C, suggesting a better discrimination for Lp(a) but not LDL‐C regardless of the presence of VHR (Figure S4), the corresponding C‐statistics of Lp(a) versus LDL‐C were 0.565 versus 0.515 in overall patients, 0.585 versus 0.502 in VHR subgroup, and 0.544 versus 0.534 in non‐VHR subgroup, respectively.

## 3 DISCUSSION

With the renewal of the knowledge of ASCVD, the finer division of risk stratification has been updated in current cholesterol guideline.^12^ However, little attention was paid to the association between Lp(a) levels and the subgroups of ASCVD with different risk stratifications. Our data first established the relationship between Lp(a) and current risk stratification of ASCVD and highlighted the important role of Lp(a) in patients at different risk stratification for risk of recurrent CVEs, suggesting that the higher the risk stratification, the better the correlation of Lp(a) and CVEs. First, there was an upward trend in Lp(a) levels across the current guideline‐refined risk groups of ASCVD, indicating that circulating Lp(a) might be higher in patients with a higher risk stratification. Second, there was a significant increase in the incidence and risk of CVEs regardless of other risk factors in patients who had high Lp(a) or risk stratification, regarding Lp(a) in combination with ASCVD risk stratification in clinical management. Third, the value of Lp(a) elevation for CVEs risk prediction was evident in patients at VHR but not those at non‐VHR. The measurement and management of Lp(a) may be more valuable and a lower Lp(a) cutoff value may contribute to further decrease of CVEs risk in those patients with VHR compared with patients at non‐VHR.

Lp(a), which has the highest polymorphism, composed of LDL‐like particle containing one molecule of apolipoprotein (apo)B‐100 covalently bound to apo(a), a glycoprotein characterized by repeats of an unusual “kringle” structure.^21^ The concentration of Lp(a) in circulation is strictly regulated by the *LPA* gene locus and predominantly genetically determined (>90%) and is relatively constant throughout life span.^21^, ^22^ Furthermore, no medications involved to effectively lower Lp(a) for our patients during the present study period. Most of the subjects were taking statins that may interfere slightly in circulating Lp(a), but the treatment does not make clinical importance.^23^, ^24^ Proprotein convertase subtilisin/kexin type 9 inhibitors (PCSK9i) have emerged as therapeutics to lower Lp(a) but were not available in the study patients on their enroll time. Therefore, Lp(a) in the present study could be considered as an untreated condition. Our novel findings indicate that an increase in Lp(a) across the distribution contributed to apparent cardiovascular risk in patients at VHR, the scenario was consistent irrespective of whether a percentile‐ or concentration‐zone used to define Lp(a). Particularly, even an incremental growth in the normal range of Lp(a) levels was accompanied by a substantial increase in risk of CVEs among VHR patients. These findings are not only relevant in the context of current recommendations regarding on‐time measurement of Lp(a) but also support for strict Lp(a) management on patients at a higher risk grade for further cardiovascular benefit. However, we did not observe significant difference in HRs between patients with high‐ and low‐Lp(a) levels among non‐VHR patients. Considering the variability in Lp(a) levels among various races/ethnicities, the results may not generalize to other settings and the association of Lp(a) with long‐term incident ASCVD risk in this subgroup of patients remains to be investigate. As far as we know, similar results were observed regarding of Lp(a) with other phenotypes of atherosclerosis. For example, Mehta et al.^18^ revealed that elevated Lp(a) stratify ASCVD risk among patients with coronary artery calcification (CAC), a marker of atherosclerosis, but not in those without CAC. Another study from Kaiser et al.^19^ showed that high Lp(a) was associated with the progression of low‐attenuation plaque phenotype undergoing repeat coronary computed tomography angiography but no difference in the progression of these more stable plaque phenotypes between high‐ and low‐Lp(a) groups. Our results offer additional evidence supporting for determining Lp(a) to identify patients at severer risk and may agree with that the identification of Lp(a) is important for better tailor the risk‐reducing strategy among ASCVD patients, particularly VHR.

However, several potential limitations of the present study require closer attention. First, this is a single‐center study with a cardiovascular specialty nature, the enrolled patients were mainly those with CAD, the number of individuals suffered from other ASCVD events were relatively small. Generalizability and representativeness of the present findings to other populations may be limited. Second, data were ascertained through medical records with privacy and capacity restrictions for some conditions. For example, FH was considered according to clinical diagnosis rather than genetic testing, and we were not able to enlarge the sample size and extend the length of follow‐up. Third, Lp(a) levels are known to vary with race/ethnicity and lack of standardization in the biochemical evaluation. The genetic determinants of elevated Lp(a) may help discern the risk of Lp(a)‐associated ASCVD that is not always conclusive from plasma measurement alone.^22^ The present study only focused the circulating Lp(a) levels but not put their genetic risk in consideration and the evaluation of the dynamic variability during the follow‐up process was missed. PCSK9i as therapeutics to lower Lp(a) concentrations were not marketed or universally accessible in China for the duration of the present study. Future studies in the PCSK9i era of Lp(a) posttherapy to fully define the risk for ASCVD, particularly VHR‐ASCVD, would be valuable. Finally, the current analyses focused on the association between Lp(a) and CVEs at different risk stratification and did not establish a conventional prediction model. The accurate value of Lp(a) and risk stratification of ASCVD in recurrent CVEs prediction may acknowledge the need to further examine and reappraise.

In conclusion, the present study first reveals the relation of Lp(a) plasma levels to the current guideline‐refined risk stratification of ASCVD. Lp(a) might be regarded as a lipid component worse than LDL‐C and presented dissimilarity in risk of CVEs among patients at different risk stratification of ASCVD. The value of Lp(a) elevation, even within the normal range, on CVEs prediction was evident in patients at VHR but not in those at non‐VHR. Our findings replenish the knowledge of current ASCVD refinement and Lp(a) in combination for CVEs prediction among a cohort of Chinese patients. Altogether, refining risk stratification for ASCVD is crucial for association evaluation and optimal prevention guidance, Lp(a) might contribute more to CVEs risk in VHR subgroup than non‐VHR of ASCVD.

## 4 SUBJECTS AND METHODS

### 4.1 Study design and patients

From April 2011 through July 2018, there were 9944 adults with confirmed ASCVD as discussed earlier in the present observational study with a prospective design.^25^, ^26^ Medications at enrollment including antiplatelet drugs (aspirin and/or clopidogrel), angiotension converting enzyme inhibitors/angiotonin receptor blockers (ACEI/ARB), β‐blockers, and statins were collected. The source of the cohort was the patients hospitalized in our division named the Cardiometabolic Center of FuWai Hospital, which is the largest cardiovascular center in China that admits patients from all over the country.

### 4.2 Blood sampling and analysis

Samples of venous blood were to be collected in ethylenediaminetetraacetic acid‐treated tubes from participating subjects following a minimum of 12 h of fasting. Levels of triglyceride, LDL‐C, and high‐density lipoprotein cholesterol were determined by an automatic biochemistry analyzer (Hitachi 7150; Japan) in an enzymatic assay. Glucose levels were determined by enzyme hexokinase assay, and Tosoh Automated Glycohemoglobin Analyser (HLC‐723G8; Tokyo, Japan) was used to determine hemoglobin A1c. The other relevant biochemical and hematological parameters were measured by standard tests.

Specially, the quantification of Lp(a) was evaluated by immunoturbidimetry method [LASAY Lp(a) auto; SHIMA laboratories] and expressed in mg/dL with a normal range of  <30 mg/dL. While the assay was not associated with apo (a) isoforms, the assay was calibrated by means of an Lp(a) protein‐verified criterion, together with World Health Organization/International Federation for Clinical Chemistry and Laboratory Medicine International Reference Reagent. Intraassay CV was <10%. Compared with other populations, Lp(a) of the Asian population is lower and studies from China further confirm an Lp(a) cutoff value of 30 mg/dL instead of 50 mg/dL.^10^ Recent recommendations suggest that rather than absolute values, clinical guidelines should consider using risk thresholds with “grey” zones to either rule‐in or rule‐out cardiovascular risk.^8^ Nevertheless, still debated is whether the cut‐offs or “grey” zones of Lp(a) correlate with ASCVD risk restricted to those at extremely high levels or throughout the distribution. Therefore, we assessed Lp(a) as groups stratified by its concentration (<30, 30−50, 50−75, ≥75 mg/dL) and percentile zones (<25th, 25−50th, 50−75th, 75−90th, ≥90th) to study the relationship in each refined subgroup of ASCVD.

### 4.3 Follow‐up and endpoints

Follow‐up information was collected at the outpatient clinic or through telephone calls if necessary with every 6 months. An independent clinical event committee was composed of highly trained nurses and cardiologists who did not know about clinical, angiographic data, and the purpose of this study. We followed‐up the patients until the study end date (February 26, 2019, with a window period of 30 days). The hard endpoints were defined as recurrent events including cardiovascular death, nonfatal MI, HF, and stroke. All events records were independently reviewed to assess vessel‐related clinical events. A total of 161 cases (1.6%) were lost of the follow‐up.

### 4.4 Statistical analysis

The data were analyzed using SPSS version 26.0 software (SPSS Inc., Chicago, IL, USA). All probability values were two sided; *p* values < 0.05 were deemed to be statistically significant. Categorical variables were evaluated by numerical values and relative rates (percentages), while continuous variables were expressed as mean with standard deviation or median with IQR, as appropriate. Categorical variables are represented as number (percentage) and analyzed by chi‐squared statistical test. Differences among groups were measured with either an independent sample *t*‐test or nonparametric test if applicable. Prediction of CVEs during the follow‐up period was assessed by Cox proportional risk analysis with HRs and 95% CIs. The adjusted variables including age, sex, body mass index (BMI), systolic blood pressure (SBP), DM, LDL‐C levels, current smoking, and medications at enrollment were input into the multivariable models.

R software version 4.2.3 was used to generate the Kaplan–Meier curves, compared ROC curves and RCS. The patients were then divided into the training and validation cohorts with a ratio of 7:3 to ensure that outcome events were distributed randomly between the two cohorts. Indices of risk reclassification and discrimination, C‐statistics in ROC, was used. Statistical significance was considered at *p* < 0.05.

## AUTHOR CONTRIBUTIONS

L. S., G. Y. L., and L. J. J. conceived, designed, and programmed the study. L. S. analyzed the data and wrote the manuscript. G. Y. L. and L. J. J. reviewed, revised, and finalized the manuscript. L. H. H., Z. Y., Z. M., Z. H. W., Z. C. G., W. N. Q., X. R. X., D. Q., Q. J., and D. K. F. collected the data and consulted on the analyses. All authors have read and approved the final manuscript.

## CONFLICT OF INTEREST STATEMENT

The authors declare that there is no conflict of interest.

## ETHICS STATEMENT

This study complied with the Declaration of Helsinki and was approved by the Ethics Committee of the Fu Wai Hospital and Cardiovascular Institute, Beijing, China (approval number: IRB2012‐BG‐006). Informed written consent was obtained from all patients enrolled in this study.

## Supporting information



Supporting Information

## Data Availability

The datasets used and/or analyzed during the current study are available from the corresponding author on reasonable request.
