# Supplementary material for: Prognostic role of lipoprotein(a) in atherosclerotic cardiovascular disease risk from a perspective on current risk stratification
Source: MedComm (2020). 2024 Oct 31;5(11):e773. doi: 10.1002/mco2.773 (PMC11527816; doi:10.1002/mco2.773)

**Title:** Prognostic role of Lipoprotein(a) in atherosclerotic cardiovascular disease risk from a perspective on current risk-stratification

**Running title:** Lp(a) and ASCVD risk-stratification

**Authors:** Sha Li^1^, PhD; Hui-Hui Liu^1^, PhD; Yan Zhang^1^, PhD; Meng Zhang^1^, MD; Hui-Wen Zhang^1^, PhD; Cheng-Gang Zhu^1^, PhD; Na-Qiong Wu^1^, PhD; Rui-Xia Xu^1^, PhD; Qian Dong^1^, MD; Jie Qian^1^, PhD; Ke-Fei Dou^1^, PhD; Yuan-Lin Guo^1^*, PhD; Jian-Jun Li^1^* MD, PhD

**Affiliation:** ^1^Cardiometabolic Center, State Key Laboratory of Cardiovascular Disease, FuWai Hospital, National Center for Cardiovascular Diseases, Chinese Academy of Medical Sciences, Peking Union Medical College, BeiLiShi Road 167, Beijing 100037, China

***Correspondence:** Professor Jian-Jun Li and Yuan-Lin Guo. State Key Laboratory of Cardiovascular Disease, Cardiometabolic ward, FuWai Hospital, National Center for Cardiovascular Diseases, Chinese Academy of Medical Sciences, Peking Union Medical College, BeiLiShi Road 167, Beijing 100037, China. **Tel:** 86+10+88396077; **Fax:** 86+10+88396584; **E-mail:** [lijianjun938@126.com](mailto:lijianjun938@126.com) (Li JJ) or [guoyuanlin@sina.com](mailto:guoyuanlin@sina.com) (Guo YL)

**ORCID:** [https://orcid.org/0000- 0003-2536-4364](https://orcid.org/0000-%200003-2536-4364) (Li JJ)

**Number of tables/figures:** Tables 1; Figures 5

**Supplemental Figure**

**Figure S1** **Kaplan‐Meier survival curves across the Lp(a) index in different risk-stratifications.** (A) overall population. (B) non-VHR subgroup. (C) VHR subgroup. Kaplan-Meier survival curves compared the cumulative incidence of CVEs stratified by the Lp(a) levels at different risk-stratification of ASCVD. Lp(a), lipoprotein(a); VHR, very high risk.


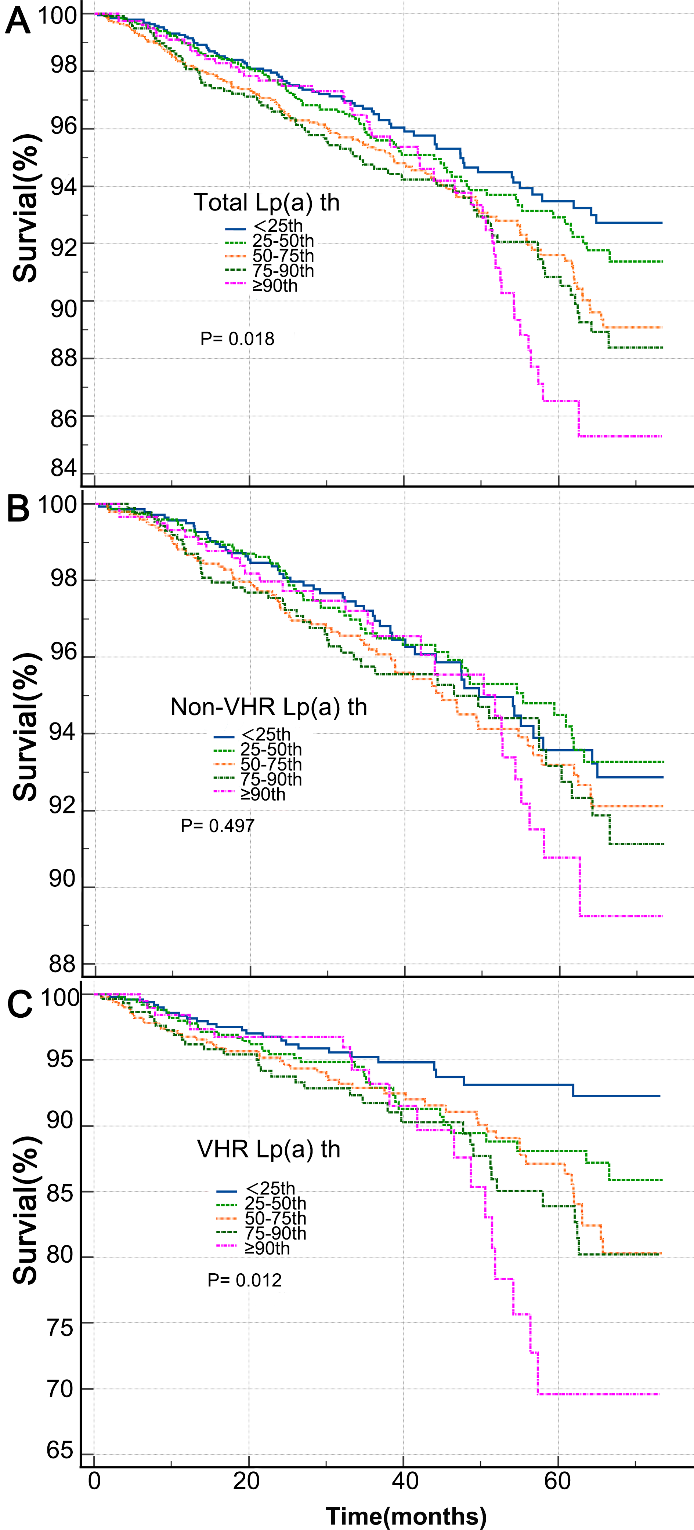


**Figure S2** **Restricted cubic spline of Lp(a) and CVEs risk.** (A) crude model in overall population. (B) adjusted model in overall population. (C) crude model in VHR subgroup. (D) adjusted model in VHR subgroup. (E) crude model in non-VHR subgroup. (F) adjusted model in non-VHR subgroup. We performed restricted cubic spline (RCS) of Lp(a) and CVEs risk. Multivariate models adjusted for age, sex, BMI, SBP, DM, LDL-C levels, current smoking and medications at enrollment. Lp(a), lipoprotein(a); CVEs, cardiovascular events; VHR, very high risk.


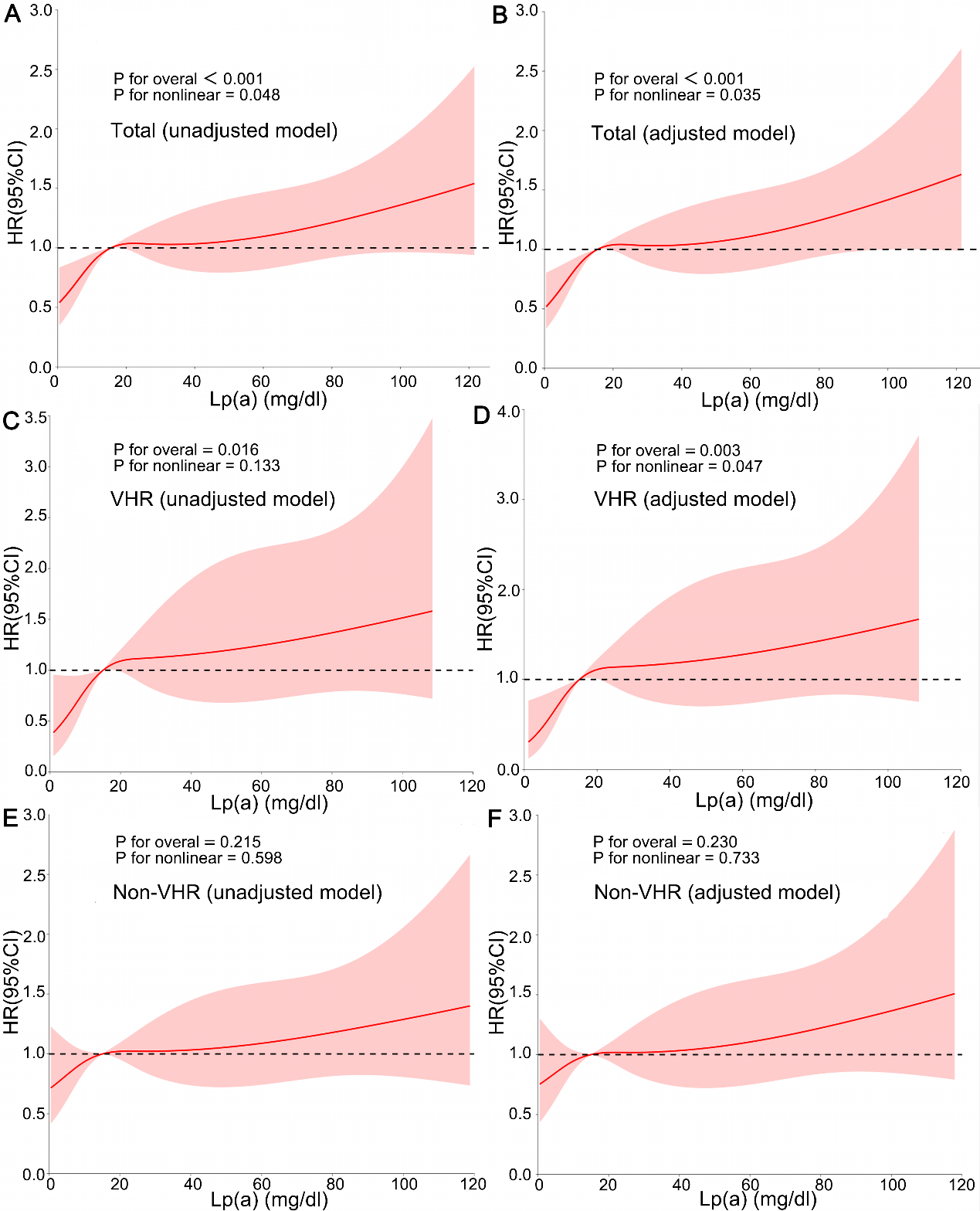


**Figure S3** **Compared receiver operator characteristic curves between VHR and non-VHR for Lp(a).** (A) overall population. (B) training cohort. (C) validation cohort. Lp(a), Compared ROC curves were showed between VHR and non-VHR for plasma Lp(a). VHR, very high risk.


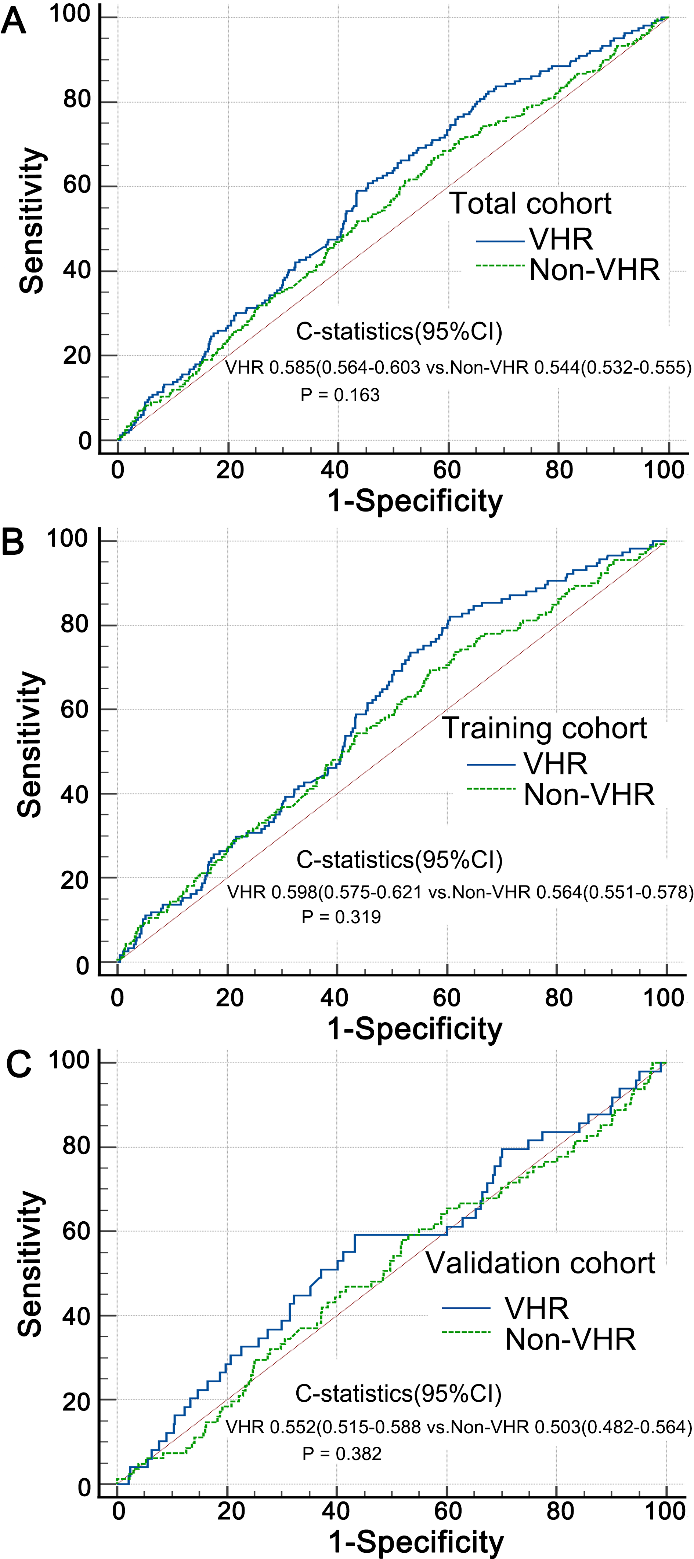


**Figure S4 Compared receiver operator characteristic curves for Lp(a) and LDL-C.** (A) overall population. (B) VHR subgroup. (C) non-VHR subgroup. Compared ROC curves were performed for plasma Lp(a) and LDL-C at different risk-stratification of ASCVD. Lp(a), lipoprotein(a); LDL-C, low-density lipoprotein cholesterol; VHR, very high risk.


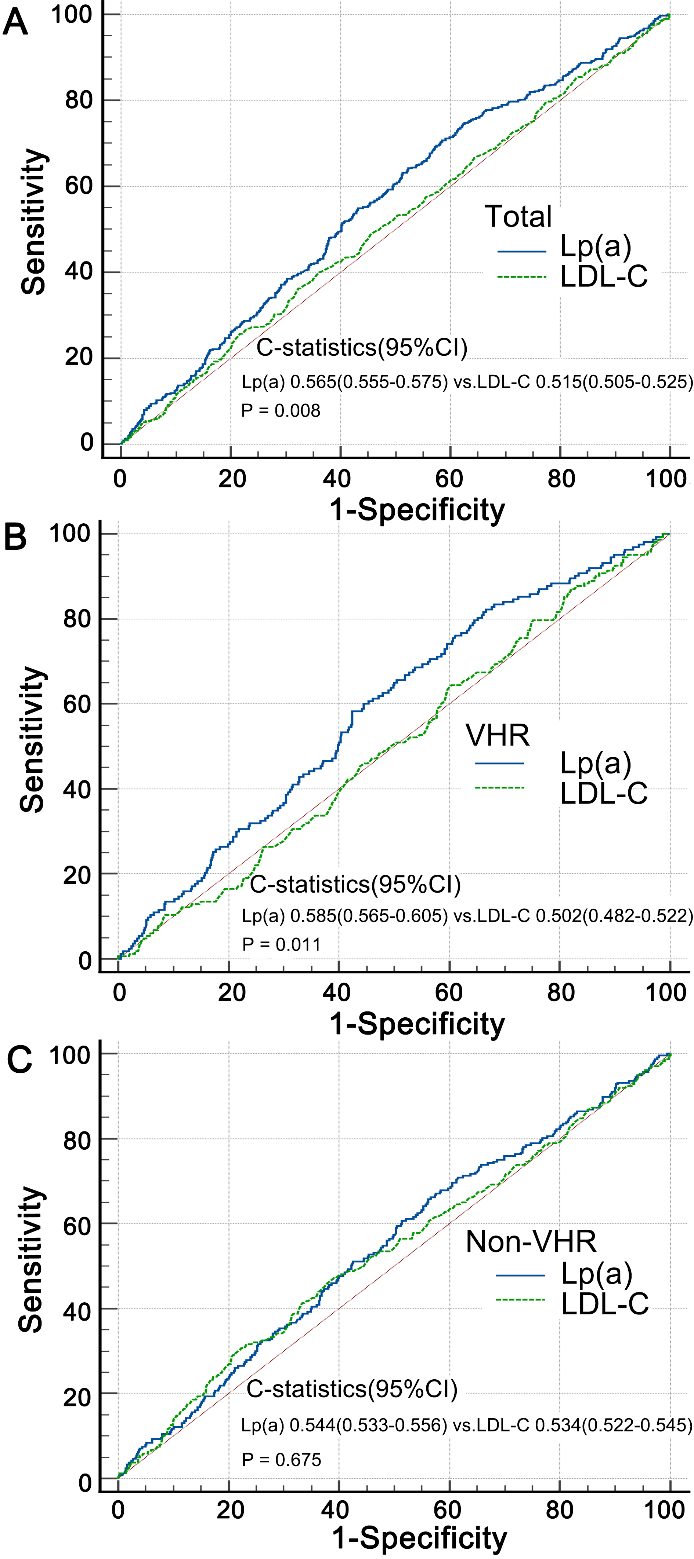

Supplement: Supplementary file 1 — Supporting Information [file MCO2-5-e773-s001.docx]
